# Bereaved parents’ perspectives of factors influencing decision-making about place of end-of-life care for children with life-limiting, life-threatening conditions: an all-Ireland qualitative study

**DOI:** 10.1186/s12904-025-01922-z

**Published:** 2025-11-24

**Authors:** Ashleen Crowe, Rachel McCauley, Yvonne Corcoran, Joanne Reid, Jayne Price, Gemma Kiernan, Eileen Courtney, Tracey McConnell, Patricia McNeilly, Veronica Lambert

**Affiliations:** 1 School of Nursing and Midwifery, Queen’s University Belfast, Belfast, Northern Ireland; 2 School of Nursing, Psychotherapy and Community Health, Dublin City University, Dublin, Republic of Ireland; 3 School of Nursing, Allied and Public Health, Kingston University London, London, England

**Keywords:** Children’s palliative care, Life-Limiting conditions, Place of End-of-Life care, Qualitative study, Decision making, Paediatric, Pediatric

## Abstract

**Background:**

The need for children’s palliative care and end-of-life services is increasing. However, there is limited evidence to inform health and social care professionals about parental preferences for place of end-of-life care for their child, or on factors influencing families in deciding on their preferred place of death for their child. The aim of this study was to explore factors which influence parental decision-making in relation to place of end-of-life care for children with life-limiting, life-threatening conditions, with respect to experiences of end-of-life care in different settings (i.e., home, hospital, hospice).

**Methods:**

This is a qualitative study. Semi-structured interviews were conducted with 20 bereaved parents (14 mothers and six fathers) of children with life-limiting, life-threatening conditions. Parents were purposively recruited from six sites inclusive of hospital, home and hospice settings across the Republic of Ireland and Northern Ireland. Interviews were digitally recorded, transcribed verbatim and thematically analysed.

**Results:**

One overarching theme, *“child first and foremost”*, and three sub-themes of *“ideal versus reality: home as place for end-of-life care”*; *“secure but not settled: hospital as place for end-of-life care”*; and *“home away from home: hospice as place for end-of-life care”* were developed.

**Conclusions:**

Selecting a place of end-of-life care and death for a child is a complex and challenging decision for parents. Important factors which influence parental decision-making around choosing home, hospital, or hospice for a child’s place of end-of-life care included: considering what would be best for their child; home was most often the first preference for parents, but this could change; that hospital was often the default choice due to lack of communication with parents about their options; and the family unit as a whole, including siblings, was an important factor when parents were making the decision. Our findings highlight that an individualised approach to supporting family’s decision-making about place of end-of-life care for their child is required. Early advance care planning could be utilised as an opportunity to begin these conversations.

**Supplementary Information:**

The online version contains supplementary material available at 10.1186/s12904-025-01922-z.

## Background

Advancements in medical technology, treatments, and research over the last two decades have resulted in increasing survival of children with life-limiting, life-threatening conditions (LLLTC) [1]. While prevalence rates of children with LLLTC are difficult to predict, figures are underestimated and rising, nationally and internationally, highlighting the need for optimisation, growth and collaboration within of children’s palliative care services [2–10]. Recent research from England indicates that the number of children with life-limiting illness has increased 148% over the 16 years between 2001/2 and 2017/8 due to advancements in medical technology [7, 11]. Predications for England indicate that this incidence will rise an additional 27% between 2018 and 2030 [7], suggesting that, in the future, many more children and their families will require access to palliative care, including end-of-life (EoL) care. This aligns with global estimates that more than 21 million children worldwide will benefit annually from palliative care, with more than eight million children requiring specialist palliative care [12].

The death of a child is a devastating loss to families, and parents often experience prolonged grief for many years [13]. The circumstances surrounding the child’s EoL care are critical, as they can profoundly influence a family’s bereavement journey. Evidence suggests that when parents feel they were involved in shared, sensitive decision-making, they report higher levels of satisfaction with their child’s care and fewer symptoms of post-traumatic stress and complicated grief [14, 15]. Hence it is vital that care is both child and family centred, where sensitive decision-making is shared between the child (where possible), parents, and health care professionals [14, 15]. While many decisions are made during advance care planning, deciding where a child will receive EoL care is critically important for achieving optimal care for the child and family [16].While home is often the preferred place of EoL care for children with LLLTCs, evidence pertaining to the decision-making process when selecting a setting for EoL care for children remains inadequate [17]. It has been shown that parents, more so than clinicians, consider home to be the most appropriate place for both EoL care and death of a child [18]. However, complexities have also been reported in the literature which influence a parents’ choice of home or not for their child’s EoL care [19]. Consequently, advance care planning and timely shared decision-making among all stakeholders is critical, as choices around place of EoL care for children are often complex and challenging.

National and international policy guidelines recommend that parents and children, where possible, should hold a central role in decision-making regarding the child’s care including place of death [20–23]. This includes respecting parents’ wishes to either remain in a clinical setting or at home. A child’s death can happen in three distinct settings of home, hospital, or hospice [19], and prior literature recommends that policymakers improve EoL care in all settings [6, 21].

Though there have been calls to improve paediatric EoL care in all settings, motivations for parents’ preferences for place of EoL care for their child is poorly understood. It is known that access and service availability impact parents’ choice [24–26], and that parents consider their own capacity to provide care when deciding on place [24, 27]. However, little is known about barriers and facilitators which might impact families’ preferred place of EoL care. While national and international policies support parental autonomy to make informed and timely decisions about place of EoL care for their child [20–22, 28], further understanding of factors which influence parents decision-making when determining place of EoL care for their child is needed. This study aimed to explore factors which influence parental decision-making in relation to place of EoL care for children with LLLTC conditions, with respect to experiences of EoL care in different settings (i.e., home, hospital, hospice).

## Methods

This is a qualitative descriptive study using semi-structured interviews. The study is reported in accordance with the Consolidated Criteria for Reporting Qualitative Research (COREQ) [29], supplemental File 1. A qualitative descriptive approach was chosen because the aim was to provide a rich and comprehensive description of bereaved parents’ direct experiences, staying close to their own words and everyday events [30]. This approach is particularly well-suited for a topic with limited existing research, as it describes a phenomenon from the perspective of those who experienced it, without imposing a pre-existing theoretical framework [30]. We collaborated with two bereaved parent representatives, one from the RoI and one from NI. Their lived experience was instrumental in guiding the research, from co-creating and piloting the interview guide to providing feedback on preliminary findings.

### Participants and recruitment

Participants were bereaved parents of children with LLLTCs. Parents were purposively recruited from six sites (three in the RoI and three in NI) across hospital, home, and hospice settings. Table 1 outlines parent(s)/guardian(s) inclusion and exclusion criteria. Based on evidence balancing the ethical need to protect bereaved parents with the need for accurate recall, our inclusion criteria required that the child’s death occurred between 6 and 48 months prior to recruitment [31]. This timeframe respects parents’ emotional vulnerability while ensuring their memories of end-of-life care are still vivid. Parent(s)/guardian(s) who met the eligibility criteria were identified by nominated health and social care professionals (HSCP) who were known to the families at each recruitment site. The HSCPs’ role was to facilitate a sensitive initial contact (via telephone or email), informing parents about the study and asking if they wished to receive an information pack (cover letter, participant information leaflet, consent form and research team contact details form). This approach was taken to ensure the recruitment process was respectful of the families’ bereavement, as it utilised the existing trust between the families and the HSCPs. If parents expressed an interest in learning more about the study, an information pack was sent by post. If parents decided to take part, they returned a contact details form directly to the research team, who subsequently followed up with the parent(s) to discuss participation, consent, and arrange an interview.

**Table 1:** Parent(s)/Guardian(s) inclusion and exclusion criteria

Inclusion Criteria	1. Parent(s)/Guardian(s) of a child (< 18 years) diagnosed with a life-limiting life-threatening condition who had died. 2. Death of the child occurred no less than six months prior to, and no greater than 48 months post initial recruitment to this study. 3. Parent(s)/Guardian(s) who influenced the place of end-of-life care for their child. 4. Able to understand and speak English.
Exclusion Criteria	1. Parent(s)/Guardian(s) whose child died suddenly or unexpectedly (e.g., an accident or rapid deterioration). 2. Parent(s)/Guardian(s) who lost an adult child (i.e., >18 years). 3. Parent(s)/Guardian(s) identified as vulnerable e.g., experiencing prolonged grief or posttraumatic stress (assessed by health and social care professional).

In addition, within Northern Ireland, recruitment was also facilitated by a closed group Facebook page, which had administrative oversight from the recruiting site. A Facebook post informed group members of the study and interested parents contacted the research team directly, and if eligible, also received an information pack.

### Data collection

Data collection took place between June and September 2023, with four researchers (three in the RoI and one in NI) with qualitative research experience interviewing all parents using a semi-structured interview topic guide (see Table 2). The interview topic guide was developed from a review of the literature, expert opinions of the research team and HSCPs, and the invaluable input of two bereaved parent representatives who also piloted the guide. Minor textual changes were made to the guide following the pilot, based on the representatives’ feedback. All interviews were audio-recorded with written parental consent, transcribed verbatim with transcripts verified by each interviewer for accuracy, and pseudonymised for analysis. If both parents wished to take part, they had the option of being interviewed together or separately.

To ensure the integrity of data collection and analysis, a rigorous process of reflexivity was used. This involved self-monitoring for personal biases and emotions and using post-interview debriefing to navigate the sensitive topic and maintain professional distance. To ensure the welfare of both bereaved parents and researchers, a comprehensive distress protocol was implemented. For participants, this protocol (adapted from Draucker et al., [32]) outlined a procedure to manage potential distress, with researchers prepared to offer breaks and referrals to bereavement support. The research team was also supported through regular debriefing meetings and access to university counselling services, while a specific safety protocol was established for researchers conducting home interviews.

**Table 2:** Interview topic guide

Topic	Guiding Questions
Child’s treatment and care	Tell me about the treatment and care your child received?
Access and experiences of EoL care	Tell me about your experiences of your child receiving EoL care services at home, in hospital, or in a hospice? In your experience, what were the most challenging/difficult aspects of your child’s EoL care that you faced as a mother/father/parents?
Decision making regarding place of EoL care	As a mother/father/parents, were you and (child’s name) provided with options/involved in the decision about place of EoL? What factors influenced your decision about where (child’s name) would receive EoL care? Who do you think should be the ultimate decision-maker about where a child receives EoL care? Do you have any suggestions about what may have improved the overall experience of EoL care for (child’s name)?

### Data analysis

Data analysis was guided by Braun and Clarke’s reflexive approach to thematic analysis [33]. The six steps were data familiarisation, data coding, generating initial themes from codes, reviewing and developing themes, defining, naming and refining themes, and writing up the report. NVivo 14 was used to assist with data management. The first interview transcript was coded independently by the first and third author (AC, YC). Both authors then checked coding for agreement to manage any potential subjectivity and ensure coding accuracy through consensus building. Next, a joint codebook was developed collaboratively and reflexively. Then the first and second author (AC, RM) continued with the coding process, each coding interview transcripts independently for each geographic region. After coding every third transcript both authors (AC, RM) checked coding agreement to ensure consensus was occurring and updated the joint codebook continuously. This process was overseen by senior researchers (YC, JP, JR) and followed until coding was completed across all interviews. Codes were then collated for the entire dataset. Next, themes were iteratively developed from the codes by identifying patterns in the dataset. Theme interpretations were refined and validated through discussion with the wider research team to ensure these accurately reflected coded extracts and content of the original interview dataset.

### Findings

Nineteen interviews were conducted with 20 bereaved parents, which included a single parent and a legal guardian. The interviews were with bereaved parents of 15 children (aged < 1–16 years) with LLLTCs who had died in hospital (*n* = 6), home (*n* = 7), and hospice (*n* = 2) settings. In most cases, mothers participated (*n* = 14). In five families, both parents participated with one couple opting to be interviewed together, and four couples opting to be interviewed separately. All interviews took place face-to-face at a time and place of the parent’s choosing. Most interviews took place in the family home (*n* = 16), with three taking place in alternative venues chosen by the participants. The interviews ranged in duration from 26 to 93 min. Demographic characteristics are shown in Table 3.

One overarching theme, *“child first and foremost”*, and three-subthemes of *“ideal versus reality: home as place for end-of-life care”*; *“secure but not settled: hospital as place for end-of-life care”*; and *“home away from home: hospice as place for end-of-life care”* were developed.

**Table 3:** Demographic characteristics of parents/guardians (*n* = 20) and their child (*n* = 15)

Demographic characteristic	Number
Parent participant • Mother/Guardian • Father/Guardian	*N* 14 6
Place of child’s death • Hospital • Hospice • Home	*N* 6 2 7
Parent resident in NI or RoI • NI • RoI	N 9 11
Age of child at end-of-life/death • < 1 year • 2 years • 4 years • 6 years • 8 years • 9 years • 12 years • 15 years • 16 years	N 4 3 1 1 1 1 2 1 1
Child’s life-limiting, life-threatening condition • Non-malignant life-limiting conditions • Cancer • Cardiac	N 11 3 1

### Overarching theme: child first and foremost

In all parents’ accounts of decision-making regarding place of EoL for their child, the overarching theme was *‘child first and foremost’*. Child first and foremost represented the extraordinary efforts parents went to, at a very challenging time, to attend to wishes of their child (either voiced by the child or perceived by parents as advocate decision maker for the child) and meet their care needs.

*“I tried to manage [son]’s care [at home] for a long time… And I just got to the point where I just*,* I don’t know if this makes sense*,* but it’s like your nervous system goes*,* just really can’t function properly. And I just knew… I can’t look after him properly*,* you know. So… they offered us hospice” (BP1*,* mother)*.

Parents revealed that when their child expressed a preference or feeling about a setting, this was an overriding influencing factor in their decision-making. Ultimately, parents were trying to do what was best for the child while having to decide where their child was to receive EoL care - a decision that no parent wanted to make.

*“Going to your daughter and think*,* “You want me to make that decision?” […] There’s a million and ten thoughts going through your mind*,* you just don’t know. And even afterwards*,* it’s an unnatural thing and it’s a thing that I can’t forgive myself for doing because you’re not supposed to make those decisions…” (BP5*,* mother)*.

Parents desired support in their parenting role from HSCPs so that they could spend as much time with their child as possible and place the needs and wishes of their child to the forefront even if this was challenging for themselves.

*“We wanted to be there because she wasn’t here for very long. So*,* we just tried to go down every day. I think I had one or two days off where I didn’t go down. Just to recuperate because it was exhausting.“ (BP16*,* mother)*.

There were times when parental choice of place of EoL care could not be met, and in these instances, parents often felt frustrated and overwhelmed by limited services and supports, as then they were unable to do what they felt was best for their child. Additionally, sometimes parents did not feel equipped to make informed decisions as information from HSCPs was often not transparent or timely which meant that their child’s place of EoL care was by default and not of parents’ choosing.

* “We were essentially sort of left on our own… [The consultant] had mentioned perhaps [name of hospice] or the [name of children’s hospital] but home was never mentioned to us… we weren’t aware of. the potential issue… in terms of very limited support if you wanted to keep your child at home for end of life.” (BP10*,* mother)*.

The three sub-themes of *“ideal versus reality: home as place for end-of-life care”*; *“secure but not settled: hospital as place for end-of-life care”*; and *“home away from home: hospice as place for end-of-life care”* informed this overarching theme and are interconnected by parents putting the child and family unit above all other considerations when making decisions about the place of their child’s EoL care.

### Sub-theme 1: ideal versus reality: home as place for end-of-life care

This sub-theme, *‘ideal versus reality: home as place for end-of-life care’* represents the gap between parents’ strong desire for their child to receive EoL care at home and the practical realities that influenced this complex decision. While home was often considered the ideal place or first option, driven by a core belief in putting the *‘child first and foremost’*, as well as a focus on what was best for the family unit, parents’ ability to realise this ideal depended on external factors. Parents relied on HSCPs for knowledge and advice needed to make this time-sensitive decision, and the availability of this information often determined whether home care was a viable reality or unachievable wish.

Being a place of familiarity, home was where all family members could feel at ease and comfortable. Being at home enabled parents to be central to decision making about their child, allowed time for the family to be together and ensured a level of continuity of family life at a very difficult and emotional time. However, it was evident that EoL care at home required appropriate preparation and ongoing support for families. Information from HSCPs was necessary to prepare parents for what to expect when EoL care was provided at home. Without adequate communication between parents and HSCPs on what EoL care at home would be like, EoL care may not have been as the parents imagined.

*“She [HSCP] said*,* you do understand that it’ll be a huge undertaking for you to have [daughter] home. I’m not saying you can’t have her home and that’s not the right thing to do*,* but I’m just giving you the full picture. She says*,* you’ll not have a nurse with you 24/7. You know*,* you will be left – not left*,* but you know*,* you’ll be doing the majority of this on your own.” (BP6*,* mother)*.

Receiving information in a timely and transparent manner about what their child’s EoL care and death would be like at home was critical when deciding where EoL care for their child would take place. While parents desired the familiarity and comfort of home, they were not familiar with healthcare practicalities, services and supports that would be required for their child at home. Parents revealed that this information was often not provided, or it was received too late to inform their decision. This resulted in some parents’ feeling let down and frustrated as their choice of home for place of EoL care was not possible. It was clear that timely information about options, and pros and cons of these options, was vital to inform parent’s decision-making about EoL care at home or not.

*“It’s getting that information you know. … say you mention end of life care and you [say] your GP could be involved and nurses can come to your house. But you’re not making it specific to what my child needs. And you know as I say*,* it’s getting you know what are the options on the table and being really honest about the pros and cons of those options. Because you can’t make the decision you know… You need to tell us the full story as hard as that may be*,* as knowing that means we’ll be going down the end-of-life care. We still need to know that you know.” (BP2*,* mother)*.

Parents spoke about how familiarity of environment, and comfort and contentment, that both the child and parents derived from being at home, close to family and friends, was a key factor in deciding on home as place of EoL care.

*“I’d say that the home surroundings*,* unless they’re in savage pain*,* the home surroundings that’s the ideal thing. Because it gives you peace of mind too. There’s familiar things*,* familiar smells are all there. That’s a great sign of contentment.” (BP4*,* parental guardian)*.

Parents revealed that when their child expressed a preference to be at home, in their familiar surroundings, this was an overriding influencing factor in their decision-making to choose home.

*“I asked [child’s name] what did he want to do. He said he wanted to go home. And it was always about home for [child’s name]*,* like [child’s name]*,* we had taken [child’s name] to the most amazing places around the world and it was always about coming home. And he said he wants to go home.” (BP13*,* mother)*.

Home also provided parents with the freedom to choose who could visit and be part of the end of their child’s life, and to maintain normality within the family unit for as long as possible. This freedom was comforting for parents and provided them with a sense of control at a time that otherwise seemed out of their control.

The decision for EoL care at home was not without its challenges and parents identified some additional factors that informed their decision. For example, other children’s presence and available space within the family home impacted on the freedom to deliver EoL care at home. Parents also worried that the experience of observing a sibling’s EoL care at home would be too distressing for their other children.

*“I was worried about traumatising [sibling’s name] because it’s a lot … and we also didn’t have a very big house then. We were in a small two-bedroom apartment*,* and it was squashed because we were saving up for this house. So*,* we weren’t in a position to really bring her home as well.” (BP16*,* mother)*.

### Sub-theme 2: secure but not settled: hospital as place for end-of-life care

This second sub-theme *‘secure but not settled: hospital as place for end-of-life care’* captures the paradox parents experienced when their child received EoL care in a hospital setting. This decision, while often a default or necessary choice, was still driven by the overarching theme of *‘child first and foremost.’*

For some parents, even though home was the preferred place of EoL care for familiarity and freedom reasons, it was not always possible. Factors such as lack of some service provisions locally to the family home, the child’s medical needs, and instability of the child’s condition, meant that home was not feasible as a preferred place for EoL care and hospital was the only option.

*“She couldn’t have been moved out of the room. So*,* the only plan we had was to leave her in the hospital […] she was on all these machines; she couldn’t have been moved either way. So*,* the only logical plan [was hospital].” (BP14*,* mother)*.

While families took comfort in the professional care and felt a sense of security in the hospital’s ability to support their child’s medical needs, they often felt unsettled by the environment itself. The hospital allowed parents to focus solely on being a parent, free from the distractions of home responsibilities, but this sense of security was often compromised by factors that created uncertainty and discomfort.

Hospital was often chosen by parents as the place of EoL care when the child was already an inpatient in hospital, when parents and their child had established relationships with HSCPs and other multidisciplinary team members in the acute hospital setting, or when there was a sudden or unexpected deterioration in the child’s condition. Parents took comfort and felt secure in professional care that hospital staff provided.

*“So many of the doctors*,* nurses*,* the cleaning staff*,* just literally every single person that has to do with the hospital knew [daughter] and knew her very well*,* so we wanted an open policy*,* and we wanted her end of life in the hospital itself*,* yeah*,* which was beautiful. It ended up being really beautiful.” (BP5*,* mother)*.

Some parents highlighted that deciding to have their child’s EoL care in hospital allowed parents, and siblings, to separate memories of death from home life. By choosing EoL care away from home parents sought to protect the dying child’s siblings from potentially distressing memories, thereby the hospital provided safety and security for siblings as well as the child themselves.

Additionally, EoL care in hospital reduced caring responsibilities for parents and allowed them time to focus on their role as parents. Some parents reported being in a state of “autopilot” and described HSCPs as being accommodating both in providing care to the family and planning for the family’s bereavement.

*“[HSCPs were] great because I was kind of on autopilot*,* I think. Especially at that time. And like they reminded me to take memories*,* they organised a photographer*,* and I got a lot of nice pictures because of them. I probably wouldn’t have done it because I was just out of that.” (BP18*,* mother)*.

However, parents also reported that hospital was often the default choice due to lack of timely, accurate and transparent communication from HSCPs regarding options for place of EoL care and advance care planning. Parents relayed that they were often trying to make decisions about what was best for their child even in the last hours, including the setting for EoL care, before their child died.

*“I don’t even think the nurses knew what [end-of-life] meant […] we ended up in the situation where we were sort of planning [end-of-life care] but then we had another instance of needing [end-of-life care]*,* and it sort of got rushed […] and then that ended up for us that happening in hospital.”* (*BP3*,* father)*.

Some parents reported a perceived lack of confidence from HSCPs in communicating about EoL care which they felt compromised the child’s safety and their own feeling of security.

*“The night before he died was horrendous*,* like the worst… But all the nurses and all the ones on that night were saying and going*,* “We can’t give him any more drugs.” I’m like he’s had the [expletive]- and then they came in the next morning and all the really senior consultants*,* all were going*,* “Why didn’t they ring us? We left explicit instruction*,*” and we’re like-… there’s a part of me don’t think even the nurse and the doctors on that night realised what palliative care was*,* would be my view” (BP3*,* father)*.

There were several environmental factors that potentially acted as a barrier to the comfort of both parents and children during EoL care in hospital. Firstly, parents reported that the busy environment of the hospital could be unsettling, increasing anxiety and distress experienced by them and their child.

*“[Child’s name] did not want to go [to hospital]*,* and she kicked*,* and she screamed*,* and she wasn’t a*,* an uncooperative child at home ever. She just did not want to do this.” (BP11*,* father)*.

Secondly, staff rotation and lack of continuity of care across the multi-disciplinary team was challenging. This difficulty was often more evident at weekends and during evenings.

*“It was clear that the nurses in [name of Children’s hospital] were under pressure. They work very long shifts. But there was some nurses in [name of Children’s hospital] that you wouldn’t expect to see back in again. And the reason they are back in is because they were short staffed*,* and they got called in. So*,* you know*,* the nursing staff were there 24 hours 7 days a week […] Monday to Friday. So*,* the place was completely different at the weekends.” (BP11*,* father)*.

Geographical distance from the family home and from sibling’s childminding facilities was another factor which influenced parent decision-making about hospital as a place of EoL care. When inpatient settings were a considerable journey away from home, parents experienced guilt over time divided between their children, distress in juggling competing demands, fear of accessing services too late, and strain and exhaustion from long journeys.

*“That was the hardest part for me. Because even coming up to the end*,* I was missing out on the other two [children]*,* and I felt like I didn’t spend as much time with [ill child’s name]. I would have done things a bit different [if I could go back].” (BP14*,* mother)*.

### Sub-theme 3: home away from home: hospice as place for end-of-life care

This sub-theme, *‘home away from home: hospice as place for end-of-life care’*, presents the factors that led parents to choose hospice as the place for their child’s EoL care. In keeping with their primary focus on putting the *‘child first and foremost’*, parents perceived hospice as an ideal middle ground. It was less clinical than a hospital but offered more support than home, providing a space where their child and family’s comfort and well-being were prioritised. Hospice was seen as a safe and nurturing environment, a true “home away from home” that provided expert palliative and EoL care in a less medicalised setting.

In placing the *‘child first and foremost’*, parents who chose hospice as the place of EoL care reported a visible reduction in distress in their child versus being in hospital.

*“[Son] really did not like hospitals like he was really scared*,* you know. It’s not a comforting environment. You know*,* you have people coming in*,* lights on*,* you know*,* it’s just not like setup for comfort [unlike hospice]. It’s set up to go in and get your treatment” (BP1*,* mother)*.

Parents also reported that their and their child’s individual and holistic needs were considered and understood by welcoming hospice staff, who provided care to the entire family in comfortable surroundings which provided a sense of ease and calm in what was a very turbulent time.

*“They were lovely at the hospice – like they are amazing and lovely […]even like bring you down your dinner and everything […] They do have a lovely communal like area and everything up there” (BP8*,* mother)*.

Parents who chose hospice described their existing supportive relationships with staff, and that they and their child had availed of respite and short break hospice services previously. This meant that both the family and child had an enduring relationship and were known in the hospice setting, seeing it often as a home away from home.

*“They just*,* everybody knew them. Everybody knew him and everybody knew. And he knew them. And I think it was that idea of this environment that like apart from being at home*,* he felt most comfortable […] And I just think at the end of his life*,* he deserved to have just his family around him you know. He deserved to have people*,* those nurses that he knew by name. And I just think he deserved that.” (BP1*,* mother)*.

Another factor that influenced parents’ decision on hospice as place for their child’s EoL care was length of time to death. In one family situation, parents spoke about the limited time they expected to have with their daughter. While preference for place of EoL care for their child was home, the shortened time to death meant hospice was an acceptable choice in this circumstance.

Parents also saw it as beneficial that they were able to control and manage who would be there during EoL care. This had value to them and added to the feeling of being supported in the hospice environment.

*“The grandparents were allowed into the hospital on the day she was born. And then when we went to hospice*,* we were wrecked and didn’t want anyone near us*,* I just can’t. Because they’re all really emotional as well because they knew it was going to happen.” (BP8*,* mother)*.

The clinical support and expertise available in hospice were also identified as important factors in choosing hospice. Within the hospice environment, parents discussed how there was expert clinical support for their child, and expert EoL support and information for them as parents. The contrast between type of care provided in hospital versus hospice contributed to parents choosing hospice, where they felt care their child and family received was less medicalised.

*“It [hospital] was very medical […] the hospice was completely different*,* you know*,* and that’s why I thought*,* no*,* I’ll do the advance care plans from the hospice because they would take [daughter] as a person and us as a family and not as medical*,* if that makes sense.” (BP6*,* mother)*.

Parents perceived hospice as a place that reduced challenges of providing EoL care at home, including attending to household activities such as cooking, washing, and cleaning.

*“I was a bit naive to think that taking her home was probably going to be the right decision. You know*,* and I’m glad that I made the decision… we made the decision to go to hospice.” (BP6*,* mother)*.

One obstacle to accessing hospice care was lack of services available, or lack of knowledge and communication about services available. Due to lack of hospice services across geographical areas, parents were often left to choose between home or hospital. Hospice services may not have even been discussed. This lack of communication between parents and HSCPs, about their options, left some parents unaware of resources and support that EoL care in children’s hospices offer.

*[Interviewer:] “Did they ever even think about transferring [child’s name] to [name of children’s hospice]?”*

*“It was never discussed.” (BP13*,* mother)*.

## Discussion

The findings presented in this study provide important information towards understanding the complex factors and emotional turmoil that parents experience when deciding on the place of EoL care for their child. One overarching theme, “*child first and foremost”*, and three sub-themes were developed: *“ideal versus reality: home as place for end-of-life care”*; *“secure but not settled: hospital as place for end-of-life care”*; and *“home away from home: hospice as place for end-of-life care”*. Collectively, our findings demonstrate that parents’ decision-making is not simply about choosing a place, but about navigating the realism and practicality of the available options. The choice is often a dynamic and complex process, influenced by a delicate balance between the parents’ wishes, the child’s needs, and the logistical realities of service provision.

Our findings provide evidence which HSCPs can use to underpin shared decision-making, as they detail the factors that parents considered when deciding on the place of EoL care for their child. These included: considering what would be best for their child; that home was most often the first preference for parents, but that this could change; that hospital was often the default choice due to a lack of communication with parents about their options; and that the family unit as a whole, including siblings, was an important factor when parents were making the decision.

We found that parents want to do what is best for their child when deciding on a place for their child’s EoL care. When a child expresses a sentiment or preference for a setting, it can overrule other influencing factors in a parent’s decision-making about where EoL would take place. These findings reflect the evidence from our previous review, which found that parents will advocate for their child’s wishes when deciding on a place of EoL care for their child, and will consider the child’s condition, including symptoms, when making this decision [7]. The findings of this study also resonate with research in the broader field of decision-making within paediatric palliative care, which indicates that a parent’s expert knowledge of their child, their desire to be a “good parent”, and their desire to do the best for their child are all crucially important during medical decision-making in general for a child with a life-limiting condition [24, 34–37]. Our findings in this study also add to this knowledge base by eliciting nuances that contribute to that decision-making. For example, parents will exhaust themselves in trying to meet the needs of their child. They will put their *“child first and foremost”*, and this will take precedence over all other considerations, even their own wellbeing. This embodiment of parental love for a child can stretch a parent’s capacity, and so when a parent’s decision about place of EoL care cannot be met it can be devastating. For parents to feel involved in the decision-making process and respected in their role, their thoughts and emotions about where their child’s EoL care should take place need to be genuinely heard and considered - especially when circumstances change or when certain requests cannot be fulfilled by HSCPs - throughout all stages of decision-making and advance care planning.

Our findings revealed that parents’ initial preference for home, while often seen as ideal, was subject to change when faced with reality. It was clear that most parents’ initial preference for their child’s EoL care was home, given the familiarity and freedom this might allow. In choosing home, parents wanted what they thought their child would want and what they felt would be best for the family unit. This supports previous studies reporting on children’s EoL care, where home was the preferred place for most children and their parents [38–40]. The decision to choose home was further strengthened if their child indicated that this was their wish or if parents perceived this to be their child’s preference.

Notwithstanding this, our findings revealed that this preference for home could change. This could be because parents realised that being at home was not what they had imagined, or because parents received untimely information, information lacking clarity, or due to their child’s condition deteriorating suddenly and requiring extensive medical interventions. This supports Gaab et al.’s study reporting that many children continue to receive EoL care in an alternative setting to their preferred place due to rapid deterioration of their illness [41]. Our findings demonstrate that this alternative setting is typically the “default” setting, which is most often the site of first contact with the child when receiving a diagnosis, typically a hospital, similar to Gaab et al.’s findings [41]. Although it would be a valuable area for future research to explore why desired parental choices were not fulfilled, this study’s findings indicate that for many parents, the options were not explicitly impossible, but rather that a lack of timely information and support from professionals made a preferred choice, such as home-based care, feel unviable. Building on this, we found that timely information about options, and the pros and cons of these options, was vital to inform parents’ decision-making. Where parents had timely information, possibly within advance care planning, they could feel secure in deciding against home as the place of EoL care despite this being their initial preference. This has implications for all practitioners who support children with palliative and EoL care needs and their families. It strengthens the importance of clear and timely conversations with the child and family about their options for EoL care so they can make a balanced, informed decision, including advanced care planning. This will also help families to pivot if their preferred option becomes unattainable. A priori literature also suggests that the initiation of advance care planning can be too late due to reservations from HSCPs who are meant to facilitate it [42, 43]. Thus, this study supports the important role of HSCPs in providing accurate, timely information and the importance of initiating conversations as early as possible.

While home and hospital were the most frequently discussed settings, hospice also emerged as an important place of care. Our findings indicate that hospices were seen as a valuable middle ground for parents, offering a “home away from home” experience. This setting provided the specialised medical care and security of a hospital while retaining a sense of normalcy and comfort that felt more like a home environment. However, the realism of hospice as a viable option was also a factor, with decisions often influenced by practicalities such as geographical distance or the availability of services. This aligns with existing literature, which has identified these logistical challenges as a known barrier to accessing paediatric palliative and hospice care [44, 45]. Parents in this study who used hospice services appreciated the holistic support, including bereavement care and opportunities for their children and family to participate in recreational activities, which helped reduce the feeling of being overwhelmed.

Parents also highlighted consideration of the needs of siblings and the wider family unit as important factors influencing their decision about place of EoL care for their child. Parents sought to select a place for EoL care where the continuity of family life could be sustained, even if this was challenging at times. They worried about how their other children would cope, especially when some parents had to spend long periods of time away from the family home and their other children. This was an additional burden on parents. Our findings demonstrated a paradox of emotions, some parents indicated that they chose a hospital for their child’s EoL care to separate memories of death from the home environment, especially for their other children, whereas for other parents, choosing home ensured the continuity of family life and sibling relationships. Building on existing literature [46, 47], our findings indicate that parents are not only aware of the emotional strain and feelings of neglect their other children may face during a sibling’s palliative care, but they also actively factor this into their decision-making. The involvement of siblings in discussions about place of EoL care would have value in potentially reducing the burden on parents who are faced with making complex decisions. When a child has a LLLTC, siblings desire communication about the condition; they want to talk about it and how it will affect their lives [47]. Literature also indicates that siblings often have an awareness of the imminent death of their siblings beyond their parents’ estimation and could be included in the decision-making process with their parents and the child who is unwell [40, 42, 49–51]. However, in cases where the siblings do not understand that death could be imminent, more information and inclusion would have value in preparing them [48]. It is also known that siblings of children receiving palliative care are often involved in the practical care needs of the child, alongside the parents, and thus are innately involved already [46]. It would seem appropriate then to include them in discussions when decision-making about location of care is taking place. Further research is required to understand how to effectively involve siblings in decision-making about a child’s EoL care, including exploring parental preferences and concerns about such inclusion. This research would inform the creation of more detailed guidance for parents and HSCPs on how to facilitate these discussions.

### Limitations

While this study offers important insights, its findings should be interpreted in light of several limitations. While our diverse, all-island sample included participants from various geographic regions, it was not perfectly balanced. Specifically, both families who accessed hospice care were from NI, meaning our findings may not fully represent the experiences of those using hospice services in the RoI. Additionally, while we adopted an all-island perspective, this study was not designed to compare differences in service availability or provision between the two jurisdictions. Future research could focus on these jurisdictional differences to inform policy and practice.

A key limitation relates to our sample’s gender and family structure. The majority of our participants were mothers, which is a common challenge in paediatric palliative care research [52, 53]. Due to their limited participation, our findings may not fully capture the distinct experiences and perspectives of fathers, which may differ from those of mothers. While we did not specifically investigate intra-couple dynamics, the limited number of couples we interviewed means we could not provide a detailed account of concordance or discordance within families about these crucial decisions. In the few couples we did interview, perspectives were largely aligned, but this is an area that warrants further exploration.

Additionally, our sample included only one legal guardian and one separated parent. This prevented us from exploring the dynamics of decision-making for guardians or investigating how end-of-life decisions are navigated in families with shared or complex caregiving arrangements. This lack of diversity in our sample, particularly regarding gender and family dynamics, highlights a significant area for future research. A deeper understanding of intra-couple decision-making, as well as the perspectives of fathers, guardians, and separated parents, is crucial for developing decision-making support protocols for healthcare professionals that are responsive to the realities of diverse family structures.

Finally, the small number of parent participants whose child received palliative care in a hospice setting is a limitation. This is reflective of the low proportion of children who access palliative care in the two children’s hospices on the island of Ireland compared to those who receive EoL care at home or in the hospital [54]. Consequently, our findings may not be transferable to families supported by these services, as their experiences with the philosophy and approach of hospice care may differ from those in other settings.

## Conclusions

When deciding on the place of EoL care for a child, the considerations made by each family are wide and varied. While, there was a consensus that home, hospital, and hospice all have their own merits and drawbacks, it was clear that every family unit would benefit from transparent and empathetic communication about the realities of EoL care in each setting. An individualised approach to supporting family’s decision making, is required, and advance care planning could be utilised as an opportunity to begin these conversations in a timely manner. Parents found comfort in each of the three EoL care settings, demonstrating that no single setting is most amenable for all families. This highlights the importance of HSCPs providing clear, succinct, and unbiased information on the options available for place of EoL care during advance care plan conversations, to help inform parents’ decision making. Future research should build upon these findings by adopting a multi-perspective approach, integrating the voices of children, siblings, and HSCPs, to develop a comprehensive, theoretically grounded understanding of this complex decision-making process.

## Supplementary Information

Supplementary Material 1.

## Data Availability

The datasets generated and analysed during the current study are not publicly available to ensure data confidentiality and protect the anonymity of the research participants, but are available from the corresponding author on reasonable request.

## Abbreviations

EoL: End-of-Life
HSCPs: Health and Social Care Professionals
LLLTCs: Life-Limiting, Life-Threatening Conditions
NI: Northern Ireland
RoI: Republic of Ireland
