# Supplementary material for: Bereaved parents’ perspectives of factors influencing decision-making about place of end-of-life care for children with life-limiting, life-threatening conditions: an all-Ireland qualitative study
Source: BMC Palliat Care. 2025 Nov 24;24:294. doi: 10.1186/s12904-025-01922-z (PMC12642087; doi:10.1186/s12904-025-01922-z)
Supplement: Supplementary file 1 — Supplementary Material 1. [file 12904_2025_1922_MOESM1_ESM.docx]

**Supplementary file 1**

Tong A, Sainsbury P, Craig J. Consolidated criteria for reporting qualitative research (COREQ): a 32-item checklist for interviews and focus groups. Int J Qual Health Care. 2007 Dec;19(6):349-57.

Consolidated criteria for reporting qualitative studies (COREQ): 32-item checklist

| **No** | **Item** | **Guide questions/Description** | **Reported on**  **Page No.** |
| --- | --- | --- | --- |
| **Domain 1: Research team and reflexivity** |  |  |  |
| Personal Characteristics |  |  |  |
| 1. | Interviewer/facilitator | Which author/s conducted the interview or focus group? | Reported under data collection and in acknowledgements respectively page 5 (AC, RM, YC) and page 22 (FH). |
| 2. | Credentials | What were the researcher's credentials? E.g. PhD, MD | Not reported in the manuscript: all authors/researchers credentials were PhD |
| 3. | Occupation | What was their occupation at the time of the study? | Not reported in the manuscript: there are ten authors / researchers, working in nursing and / psychology research. |
| 4. | Gender | Was the researcher male or female? | Not reported in the manuscript for gender neutral language: all authors/researchers were female. |
| 5. | Experience and training | What experience or training did the researcher have? | Page 5 (data collection)  All researchers had previous experience in qualitative research including conducting semi-structured interviews. |
| Relationship with participants |  |  |  |
| 6. | Relationship established | Was a relationship established prior to study commencement? | Not reported in manuscript: no relationship was established with participants prior to study commencement. |
| 7. | Participant knowledge of the interviewer | What did the participants know about the researcher? e.g. personal goals, reasons for doing the research | Page 4 (method – participants and recruitment)  Participants were provided with an information pack that explained the study and this included details of the research team. |
| 8. | Interviewer characteristics | What characteristics were reported about the interviewer/facilitator? e.g. Bias, assumptions, reasons and interests in the research topic | Not reported in manuscript: the participants were provided with an explanatory statement in the study plain language statement about the research aims and objectives and details on the research team. |
| **Domain 2: study design** |  |  |  |
| Theoretical framework |  |  |  |
| 9. | Methodological orientation and Theory | What methodological orientation was stated to underpin the study? e.g. grounded theory, discourse analysis, ethnography, phenomenology, content analysis | Page 4 (method) A qualitative descriptive approach underpinned the study to provide a straight description and comprehensive summary of the direct experiences of parents, staying close to the data and everyday events. |
| Participant selection |  |  |  |
| 10. | Sampling | How were participants selected? e.g. purposive, convenience, consecutive, snowball | Page 4 (participants and recruitment)  Participants were purposively recruited |
| 11. | Method of approach | How were participants approached? e.g. face-to-face, telephone, mail, email | Page 4 (participants and recruitment)  Participants were identified by nominated health and social care professionals via telephone or email. An advertisement was also placed on a closed group Facebook page |
| 12. | Sample size | How many participants were in the study? | Page 7 (findings)  There were 20 participants in the study, 14 mothers and 6 fathers. |
| 13. | Non-participation | How many people refused to participate or dropped out? Reasons? | Not reported in the manuscript: One participant initially agreed but then did not wish to participate |
| Setting |  |  |  |
| 14. | Setting of data collection | Where was the data collected? e.g. home, clinic, workplace | Page 7 (findings)  The interviews were either conducted in the family home or in a setting of the participant’s choice. |
| 15. | Presence of non-participants | Was anyone else present besides the participants and researchers? | Not reported in the manuscript: no one else was present besides the participant and researcher. |
| 16. | Description of sample | What are the important characteristics of the sample? e.g. demographic data, date | Characteristics of the sample are reported in the manuscript on page 7 (results) and Table 3 (page 8). |
| Data collection |  |  |  |
| 17. | Interview guide | Were questions, prompts, guides provided by the authors? Was it pilot tested? | Page 5 (data collection and Table 2 page 6) provides an overview of interview questions. |
| 18. | Repeat interviews | Were repeat interviews carried out? If yes, how many? | Not reported in the manuscript: the interview was once only for each participant. |
| 19. | Audio/visual recording | Did the research use audio or visual recording to collect the data? | Page 6 (data collection)  The interviews were audio recorded to collect the data. |
| 20. | Field notes | Were field notes made during and/or after the interview or focus group? | Not reported in the manuscript: Field notes were recorded after each interview noting contextual details. |
| 21. | Duration | What was the duration of the interviews or focus group? | Page 7 (findings)  The interviews ranged in duration from 26 to 93 minutes. |
| 22. | Data saturation | Was data saturation discussed? | Data saturation was not discussed as it does not align with the values and assumptions of the reflective thematic analysis approach reported in manuscript aligned with Braun and Clarke. |
| 23. | Transcripts returned | Were transcripts returned to participants for comment and/or correction? | Not reported in the manuscript: transcripts were not returned to participants for comment and/or correction |
| **Domain 3: analysis and findings** |  |  |  |
| Data analysis |  |  |  |
| 24. | Number of data coders | How many data coders coded the data? | Page 7 (data analysis)  Two coders (AC, RM), with input from JR, YC, and JP, were involved in the data coding process |
| 25. | Description of the coding tree | Did authors provide a description of the coding tree? | The coding tree was not reported in the manuscript. |
| 26. | Derivation of themes | Were themes identified in advance or derived from the data? | Page 7 (data analysis)  Themes were derived from the data |
| 27. | Software | What software, if applicable, was used to manage the data? | Page 7 (data analysis)  NVivo 14 software was used to manage data |
| 28. | Participant checking | Did participants provide feedback on the findings? | Not reported in manuscript: participants did not provide feedback on findings |
| Reporting |  |  |  |
| 29. | Quotations presented | Were participant quotations presented to illustrate the themes / findings? Was each quotation identified? e.g. participant number | Pages 7-16 (findings) Selected participant quotations were presented with participant numbers. |
| 30. | Data and findings consistent | Was there consistency between the data presented and the findings? | Pages 7-19 (findings and discussion)  Yes, there was consistency between data presented and findings |
| 31. | Clarity of major themes | Were major themes clearly presented in the findings? | One overarching theme was presented in the findings on pages 8-9. |
| 32. | Clarity of minor themes | Is there a description of diverse cases or discussion of minor themes? | Three sub-themes were presented in the findings on pages 9-16. |
